# Supranutrition of microalgal docosahexaenoic acid and calcidiol improved growth performance, tissue lipid profiles, and tibia characteristics of broiler chickens

**DOI:** 10.1186/s40104-023-00842-3

**Published:** 2023-03-16

**Authors:** Sahil Kalia, Andrew D. Magnuson, Tao Sun, Guanchen Liu, Woo Kyun Kim, Zackary Johnson, Xin Gen Lei

**Affiliations:** 1 Department of Animal Science, Cornell University, 252 Morrison Hall, Ithaca, NY 14850 USA; 2 Department of Poultry Science, University of Georgia, Athens, GA USA; 3 Nicholas School of the Environment, Duke University, Beaufort, NC USA

**Keywords:** Broiler, Calcidiol, Growth performance, Microalgae, Tibia

## Abstract

**Background:**

Docosahexaenoic acid (DHA) and calcidiol could be enriched in chicken for improving public nutrition and health. It remains unclear if supranutritional levels of DHA and calcidiol impair growth performance or metabolism of broiler chickens. This study was to determine singular and combined effects of high levels of supplemental DHA-rich microalgal biomass or oil and calcidiol on growth performance, concentrations of triglycerides, cholesterol, and nonesterfied fatty acids in plasma, liver, breast, and thigh, and biophysical properties of tibia.

**Methods:**

In Exp. 1, 144 day-old Cornish chicks were divided into 4 groups (6 cages/treatment, 6 birds/cage), and were fed a corn-soybean meal basal diet (BD), BD + 10,000 IU calcidiol/kg (BD + Cal), BD + 1% DHA-rich *Aurantiochytrium* (1.2 g DHA/kg; BD + DHA), and BD + Cal + DHA for 6 weeks. In Exp. 2, 180 day-old chicks were divided into 5 groups, and were fed: BD, BD + DHA (0.33% to 0.66% oil, 1.5 to 3.0 g DHA/kg), BD + DHA + EPA (1.9% to 3.8% eicosapentaenoic acid-rich *Nannochloropsis* sp. CO18, 0.3 to 0.6 g EPA/kg), BD + DHA + calcidiol (6000 to 12,000 IU/kg diet), and BD + DHA + EPA + Cal for 6 weeks.

**Results:**

Birds fed BD + Cal diet in Exp. 1 and BD + DHA + EPA diet in Exp. 2 had higher (*P* < 0.05) body weight gain (10%–11%) and gain:feed ratio (7%), and lower (*P* < 0.05) total cholesterol and triglyceride concentrations in plasma (18%–54%), liver (8%–26%), breast (19%–26%), and thigh (10%–19%), respectively, over the controls. The two diets also improved (*P* < 0.05) tibial breaking strength (8%–24%), total bone volume (2%–13%), and (or) bone mineral density (3%–19%) of chickens.

**Conclusion:**

Supranutrition of dietary calcidiol and DHA alone or together did not produce adverse effects, but led to moderate improvements of growth performance, lipid profiles of plasma and muscle, and bone properties of broiler chickens.

**Supplementary Information:**

The online version contains supplementary material available at 10.1186/s40104-023-00842-3.

## Background

Biofortifications of chicken with bioactive nutrients such as DHA and calcidiol [25(OH)D_3_] have been viewed as an effective strategy to produce health-promoting meat for human consumption [1, 2]. Relatively low to moderate inclusion levels of calcidiol (1600 to 2800 IU/kg diet) [3, 4] and DHA-rich microalgal biomass or oil (0.55 to 2.55 g DHA/kg diet) [5–7] in broiler diets caused no negative effects on growth performance, lipid profile of tissues, or bone strength. In contrast, a high inclusion of calcidiol (27,600 IU/kg diet) in broiler diet decreased body weight by 5% [8], and a high inclusion of DHA-rich microalgal biomass (6.8 g DHA/kg diet) decreased growth performance by 19% and breast muscle weight by 21% [9]. However, these past studies were focused on fortifying chicken with DHA [10] and calcidiol singularly [4]. Little research was attempted to enrich chicken simultaneously with these two nutrients or to look out for potential adverse effects of a combined high supplementation of these two nutrients on growth performance, lipid metabolism, and bone integrity of chickens.

To fill in the gap of knowledge, we conducted two experiments to examine those effects of supplementing high levels of these two bioactive nutrients in broiler chickens. In the first experiment, we supplemented 1% DHA-rich *Aurantiochytrium* sp. biomass (1.2 g DHA/kg) as the source of DHA in diets for the enrichment of DHA according to our previous findings [11]. In a subsequent experiment, we used a commercial source of DHA-rich microalgal oil (1.5 to 3.0 g DHA/kg) as the source of DHA, along with an EPA-rich *Nannochloropsis* sp. CO18 biomass (0.3 to 0.6 g EPA/kg). In both experiments, we supplemented a feed grade of synthetic calcidiol (6000 to 12,000 IU/kg) in vitamin D-adequate diets (300 IU/kg) as the source of bioactive cholecalciferol.

## Materials and methods

### Animal, diets, and management

Our animal protocols were approved by the Cornell University Institutional Animal Care and Use Committee. The DHA-rich microalgal biomass (*Aurantiochytrium*, 12% DHA in the biomass) and oil (45% DHA in the oil) were provided by Heliae (Gilbert, AZ, USA) and Archer Daniels Midland Company (ADM, Decatur, IL, USA), respectively. The EPA-rich microalgal biomass (*Nannochloropsis* sp. CO18, 1.6% EPA in the biomass) and calcidiol (Rovimix HyD Premix, 138 mg calcidiol/kg of premix) were provided by Duke University (Beaufort, NC, USA) and Royal DSM N.V. (DSM, Parsippany, NJ, USA), respectively. In Exp. 1, a total of 144 day-old Cornish male broiler chicks were purchased from Moyer’s Chicks (Quakertown, PA, USA) and housed in a temperature-controlled unit at Cornell University Poultry Research Farm. Chicks were allotted into 4 treatment diets (6 cages/treatment, 6 birds per cage). Birds were fed 1 of the 4 diets: a corn-soybean meal basal diet (BD), BD + 10,000 IU calcidiol/kg of diet (BD + Cal), BD + 1% DHA-rich microalgal biomass (*Aurantiochytrium,* 1.2 g DHA/kg diet; BD + DHA), and BD + Cal + DHA. The supplemental dietary level of DHA was based on the results of our previous study in which grade levels of the same microalgal biomass were used for enriching DHA in tissues of chickens [11]. The 10,000 IU of calcidiol/kg was chosen based on the safe range of supplementations reported in literature for the enrichments of calcidiol or testing of toxicity [12, 13]. In the BD and all treatment diets, cholecalciferol was supplemented at 1.5-fold (300 IU/kg) of the recommendation by National Research Council (NRC, 1994) [14].

In Exp. 2, a total of 180 day-old Cornish male broiler chicks were purchased from same supplier as in Exp. 1 and allotted into 5 treatment diets (6 cages/diet, 6 birds/cage): BD, BD + DHA (0.33% and 0.66% of the DHA-rich microalgal oil to provide 1.5 and 3.0 g DHA/kg diet for 0–3 and 4–6 weeks, respectively), BD + DHA + EPA (1.9% and 3.8% of EPA-rich *Nannochloropsis* sp. CO18 to provide 0.3 and 0.6 g EPA/kg diet for 0–3 and 4–6 weeks, respectively); BD + DHA + Cal (6000 and 12,000 IU of calcidiol/kg diet for 0–3 and 4–6 weeks, respectively); and BD + DHA + EPA + Cal (a combination of all 3 supplements at the doses used in diets of 2 supplements). Different from the design of Exp. 1, we used the commercial source of microalgae DHA oil, with more concentrated DHA than the microalgal biomass, to provide higher supplementations of DHA for a better enrichment outcome. We also intended to determine if supplemental both DHA and EPA (at a ratio of 5:1) could enrich both in the chicken tissues. The design for doubling the supplementation of DHA, EPA, and calcidiol from the starter diet to the grow diet was for a high enrichment efficiency of these nutrients by avoiding a potential feedback or homeostatic regulation [15, 16]. Likewise, the BD and all treatment diets were supplemented with cholecalciferol at 1.5-fold (300 IU) of the NRC recommendation [14]. All other nutrients in all diets used in both experiments were formulated to meet the nutrient requirements for broilers by NRC [14]. Compositions of starter and finisher diets used in both experiments are presented in Additional file 1: Table S1–4. Both experiments lasted for 6 weeks, Birds had free access to feed and water and received a lighting schedule of 22 h light and 2 h dark throughout.

### Growth performance and sample collections

During both experiments, body weights of individual birds were recorded at week 3 and week 6. Feed disappearance of cages were recorded weekly to calculate feed intakes. Chicken health and mortality were checked daily. At the end of week 3 and week 6, 2 birds per cage were euthanized via asphyxiation with carbon dioxide. Blood was drawn from heart puncture by using heparinized needles to prepare plasma samples that were stored at –20 °C until analysis. Liver, breast, thigh, and tibia samples were removed and stored at –20 °C for later analyses.

### Laboratory analyses

Concentrations of non-esterified fatty acids (NEFAs), total cholesterol (TC), triglycerides (TGs), and phospholipids (PL) in plasma, liver, breast, and thigh samples were determined using commercially available kits (Wako Chemicals, Richmond, VA, USA) as described in previous studies [17, 18]. In Exp. 1, tibia bone (week 6) characteristics were determined following the protocol described previously [19]. Briefly, soft tissues were removed manually from the bone. The length, width, and depth were measured at the center of the shaft for both tibias and averaged for each bird. Bone breaking strength was measured on the right tibia with the use of an Instron 5965 (Instron Corporation, Norwood, MA, USA) equipped with a 5-kN load cell and a cross-head speed of 20 mm/min. Bluehill 3 Testing Software (Instron Corporation, Norwood, MA, USA) was used to perform flexure tests with a 38-mm supported length. Maximum slope, maximum load, and energy to maximum load were recorded for each tibia. In Exp. 2, characteristics of tibia (week 6) bone were determined using Micro-CT using the method described by Sharma et al. [20]. Briefly, tibia bones were thawed at 4 °C and cleaned of all soft tissues, and analyzed by Skyscan X-ray microtomography (Bruker MicroCT, Billerica, MA, USA). The X-ray source was set at 75 kV and 133 µA. The pixel size was fixed at 25 µm, the rotation angle of 0.4^o^ was applied at each step, and 4 images per rotation were captured. A series of 2D images were captured, which were later used to reconstruct a 3D image using N-Recon (Brucker MicroCT, Billerica, MA, USA). Microtomography was performed on the distal epiphyses of the tibia, and a part of the distal supracondylar region was selected as a volume of interest wherein all bone sections (cortical bone and trabecular bone) were present. Percentage bone volume and bone mineral density (BMD) were measured from the whole total volume of interest, cortical bone, and trabecular bone sections. From trabecula bone, trabecular thickness, trabecular separation, and degree of anisotropy were also measured.

### Statistical analysis

Data from Exp. 1 and 2 were analyzed by two-way (2 by 2 factorial arrangement of treatments) and one-way analysis of variance (ANOVA) using a completely randomized design, respectively. Data were presented as mean ± SEM and *P* < 0.05 was assumed to be statistically significant. Means of different treatment groups were compared using Duncan’s multiple range test. Pen was served as an experimental unit (*n* = 6).

## Results

### Growth performance

In Exp. 1, there was no difference in the body weight gain (BWG) or feed intake of chicks among the 4 treatment diets at week 3 (Additional file 1: Table S5). Compared with those fed BD, birds fed BD + Cal had 11% higher (*P* < 0.05) BWG, and 7% higher (*P* < 0.05) gain:feed ratio at week 6 (Table 1). Although birds fed BD + DHA had 6% higher BWG and 8% higher feed intake (8%) than those fed BD, the differences were not statistically significant. Moreover, birds fed BD + Cal + DHA also showed non-significantly higher BWG (9%) and feed intake (12%) compared with birds fed BD at week 6 (Table 1).

**Table 1:** Effects of supplementation of calcidiol, DHA-rich microalgal biomass or oil, and EPA-rich microalgal biomass on growth performance of broiler chickens in Exp. 1 and 2 (0–6 weeks)

Exp. 1							
**Parameters**	**BD**	**BD + Cal**	**BD + DHA**	**BD + Cal + DHA**	**SEM**	***P*-value**	
BW 0 d, g/chick	41.9	41.8	41.8	41.7	0.170	0.85	
BW 0–6 weeks, g/chick	2910^a^	3230^b^	3090^ab^	3160^ab^	51.20	< 0.01	
BWG 0–6 weeks, g/chick	2870^a^	3190^b^	3040^ab^	3120^ab^	57.25	< 0.01	
FI 0–6 weeks, g/chick	4860^a^	5050^ab^	5240^ab^	5450^b^	115.4	< 0.01	
Gain:Feed	0.590^a^	0.630^b^	0.580^a^	0.570^a^	0.008	< 0.01	
**Exp. 2**							
	**BD**	**BD + DHA**	**BD + DHA + EPA**	**BD + DHA+ Cal**	**BD + DHA + EPA + Cal**	**SEM**	***P*-value**
BW 0 d, g/chick	42.3	42.3	42.4	42.4	42.4	0.07	0.64
BW 0–6 weeks, g/chick	2520^a^	2600^ab^	2770^b^	2530^ab^	2590^ab^	62.82	0.04
BWG 0–6 weeks, g/chick	2480^a^	2560^ab^	2730^b^	2490^ab^	2550^ab^	62.84	0.04
FI 0–6 weeks, g/chick	3880^a^	3870^a^	4410^b^	4080^ab^	4470^b^	121.8	< 0.01
Gain:Feed	0.640^ab^	0.660^b^	0.620^ab^	0.610^ab^	0.570^a^	0.02	0.02

*BW* Body weight, *BWG* Body weight gain, *FI*: Feed intake

Exp. 1; BD = Corn-soybean basal diet; BD + Cal = BD + 10,000 IU calcidiol/kg of diet; BD + DHA = BD + 1% DHA-rich microalgal biomass; BD + Cal + DHA = BD + Cal + 1% DHA-rich microalgal biomass

Exp. 2; 0–3 weeks: BD = Corn-soybean basal diet; BD + DHA = BD + 1.5 g of DHA oil/kg; BD + DHA + EPA = BD + DHA + 0.3 g/kg *Nannochloropsis* sp. CO18; BD + DHA + Cal = BD + DHA + 6000 IU calcidiol/kg; BD + DHA + EPA + Cal = BD + DHA + EPA (0.3 g/kg *Nannochloropsis* sp. CO18) + 6000 IU calcidiol/kg

Exp. 2; 4–6 weeks: BD = Corn-soybean basal diet; BD + DHA = BD + 3.0 g DHA oil/kg; BD + DHA + EPA = BD + DHA + 0.6 g/kg *Nannochloropsis* sp. CO18; BD + DHA + Cal = BD + DHA + 12,000 IU calcidiol/kg; BD + DHA + EPA + Cal = BD + DHA + EPA (0.6 g/kg *Nannochloropsis* sp. CO18) + 12,000 IU calcidiol/kg

^a,b^Means bearing the different superscripts in a row differ (*P* < 0.05)

Data are expressed as means (*n* = 6 cages and 6 birds/cage) and were analyzed by two-way and one-way ANOVA in Exp. 1 and 2, respectively

In Exp. 2, birds fed BD + DHA + EPA had 17%–27% higher BWG and 6%–25% higher gain:feed ratio than those fed the other diets at week 3, but the differences were not statistically significant (Additional file 1: Table S5). Compared with birds fed the BD, birds fed BD + DHA + EPA had 10% higher (*P* < 0.05) BWG and 14% higher (*P* < 0.05) feed intake at week 6 (Table 1). Birds fed the BD + DHA diet had 3% higher BWG and gain:feed ratio, compared with birds fed BD, but the differences were not statistically significant (Table 1). Moreover, birds fed BD + DHA + EPA + Cal had 15% higher (*P* < 0.05) feed intake, compared with those fed BD. No difference in the BWG, feed intake, or gain:feed ratio was shown in birds fed BD + DHA + Cal, compared with those fed BD (Table 1).

### Plasma and tissue lipid profiles

In Exp. 1, there was no difference in concentrations of plasma TGs, TC, and NEFAs among all 4 diets at week 3 (Table 2). At week 6, birds fed BD + Cal had 22% and 29% lower (*P* < 0.05) plasma TC concentrations compared with birds fed BD + DHA or BD alone, respectively. In Exp. 2, diets produced no significant effects on any of the lipid profiles in plasma or tissues at week 3 (Table 3). However, at week 6, birds fed BD + DHA + EPA had lower (*P* < 0.05) concentrations of TGs (plasma 54%; liver 18%; breast 24%; thigh 19%), TC (plasma 18%; liver 8%; breast 19%; thigh 9%), and NEFA (plasma 12%; liver 26%; breast 26%; thigh 13%), than birds fed BD, respectively (Table 4).

**Table 2:** Effects of supplementation of calcidiol and DHA-rich microalgal biomass on plasma lipid profile of broiler chickens in Exp. 1

Item	**BD**	**BD + Cal**	**BD + DHA**	**BD + Cal + DHA**	**SEM**	***P*-value**
Week 3						
TG, mg/dL	22.5	22.2	25.3	28.1	3.24	0.65
TC, mg/dL	133	136	139	130	5.03	0.62
NEFA, µmol/mL	0.210	0.180	0.170	0.160	0.02	0.27
Week 6						
TG, mg/dL	20.3	23.5	21.8	22.5	1.97	0.55
TC, mg/dL	101^b^	78.0^a^	97.8^b^	101^b^	4.42	< 0.01
NEFA, µmol/mL	0.240	0.240	0.240	0.280	0.03	0.69

*NEFA* Non-esterified fatty acids, *TC* Total cholesterol, *TG* Triglycerides

BD = Corn-soybean basal diet; BD + Cal = BD + 10,000 IU calcidiol/kg of diet; BD + DHA = BD + 1% DHA-rich microalgal biomass; BD + Cal + DHA = BD + Cal + 1% DHA-rich microalgal biomass

^a,b^Means bearing the different superscripts in a row differ (*P* < 0.05)

Values are expressed as means of 6 birds/treatment and data were analyzed using two-way ANOVA

**Table 3:** Effects of supplementation of DHA-rich microalgal oil, EPA-rich microalgal biomass, and calcidiol on plasma and tissue lipid profiles of broiler chickens at week 3 in Exp. 2

Item	**BD**	**BD + DHA**	**BD + DHA + EPA**	**BD + DHA + Cal**	**BD + DHA + EPA + Cal**	**SEM**	***P*-value**
Plasma							
PL, mg/dL	102	90.2	91.5	101	104	6.26	0.48
TG, mg/dL	31.6	25.5	33.7	25.2	35.0	2.88	0.08
TC, mg/dL	100	103	90.6	106	107	2.50	0.41
NEFA, µmol/mL	219	204	202	230	212	15.6	0.72
Liver							
PL, mg/g tissue	16.7	14.7	15.2	16.1	15.8	2.42	0.16
TG, mg/g protein	66.8	70.1	64.8	65.1	70.0	5.17	0.95
TC, mg/g protein	15.3	14.1	15.0	14.8	14.6	1.14	0.81
NEFA, µmol/g protein	45	46	41.1	42.0	43.4	3.80	0.65
Breast							
PL, mg/g tissue	6.01	4.16	6.12	5.76	6.09	0.44	0.06
TG, mg/g protein	21.4	21.2	19.3	21.1	20.8	1.36	0.73
TC, mg/g protein	6.36	6.10	6.53	6.69	6.80	1.24	0.80
NEFA, µmol/g protein	5.27	5.12	4.85	4.96	4.79	0.70	0.56
Thigh							
PL, mg/g tissue	3.75	3.25	3.43	3.64	4.34	0.52	0.64
TG, mg/g protein	23.5	22.1	21.6	23.4	22.4	1.22	0.87
TC, mg/g protein	4.56	4.60	4.53	4.37	4.32	0.51	0.64
NEFA, µmol/g protein	5.50	5.33	5.06	5.53	5.24	0.83	0.71

BD = Corn-soybean basal diet; BD + DHA = BD + 1.5 g of DHA oil/kg; BD + DHA + EPA = BD + DHA + 0.3 g/kg *Nannochloropsis* sp. CO18; BD + DHA + Cal = BD + DHA + 6000 IU calcidiol/kg; BD + DHA + EPA + Cal = BD + DHA + EPA (0.3 g/kg *Nannochloropsis* sp. CO18) + 6000 IU calcidiol/kg

Values are expressed as means of 6 birds/treatment and data were analyzed using one-way ANOVA

**Table 4:** Effects of supplementation of DHA-rich microalgal oil, EPA-rich microalgal biomass, and calcidiol on plasma and tissue lipid profiles of broiler chickens at week 6 in Exp. 2

Item	**BD**	**BD + DHA**	**BD + DHA + EPA**	**BD + DHA + Cal**	**BD + DHA + EPA + Cal**	**SEM**	***P*-value**
Plasma							
PL, mg/dL	78.9	61.7	67.1	76.3	70.7	4.50	0.08
TG, mg/dL	40.4^b^	27.8^ab^	18.7^a^	39.2^b^	28.8^ab^	3.76	< 0.01
TC, mg/dL	102^b^	89.6^ab^	83.8^a^	90.3^ab^	79.8^a^	3.53	< 0.01
NEFA, µmol/mL	87.3^b^	62.0^a^	76.6^ab^	85.6^b^	78.8^ab^	4.21	< 0.01
Liver							
PL, mg/g tissue	14.6	12.3	12.5	14.7	13.7	1.74	0.22
TG, mg/g protein	71.4^b^	68.2^b^	58.7^a^	70.9^b^	66.1^ab^	6.12	< 0.01
TC, mg/g protein	14.7	13.3	13.6	14.1	13.6	1.37	0.95
NEFA, µmol/g protein	46.0	41.5	34.1	42.1	38.8	3.10	0.15
Breast							
PL, mg/g tissue	3.60	2.89	3.09	3.38	2.76	0.19	0.32
TG, mg/g protein	23.8^b^	22.9^b^	18.1^a^	23.1^b^	22.9^b^	2.11	0.04
TC, mg/g protein	5.82	4.61	4.69	5.41	5.59	0.30	0.05
NEFA, µmol/g protein	5.08^b^	4.67^ab^	3.78^a^	4.50^ab^	4.44^ab^	0.91	0.03
Thigh							
PL, mg/g tissue	3.34	2.47	2.92	2.83	2.56	0.32	0.12
TG, mg/g protein	26.6^b^	24.5^ab^	21.5^a^	24.2^ab^	24.0^ab^	2.16	0.01
TC, mg/g protein	4.00	3.93	3.63	3.88	3.78	0.11	0.21
NEFA, µmol/g protein	5.18	4.99	4.58	5.07	4.53	1.01	0.90

0–3 weeks: BD = Corn-soybean basal diet; BD + DHA = BD + 1.5 g of DHA oil/kg; BD + DHA + EPA = BD + DHA + 0.3 g/kg *Nannochloropsis* sp. CO18; BD + DHA + Cal = BD + DHA + 6000 IU calcidiol/kg; BD + DHA + EPA + Cal = BD + DHA + EPA (0.3 g/kg *Nannochloropsis* sp. CO18) + 6000 IU calcidiol/kg

4–6 weeks: BD = Corn-soybean basal diet; BD + DHA = BD + 3.0 g DHA oil/kg; BD + DHA + EPA = BD + DHA + 0.6 g/kg *Nannochloropsis* sp. CO18; BD + DHA + Cal = BD + DHA + 12,000 IU calcidiol/kg; BD + DHA + EPA + Cal = BD + DHA + EPA (0.6 g/kg *Nannochloropsis* sp. CO18) + 12,000 IU calcidiol/kg

^a,b^Means bearing the different superscripts in a row differ (*P* < 0.05)

Values are expressed as means of 6 birds/treatment and data were analyzed using one-way ANOVA

### Tibia bone health

In Exp. 1, tibia from birds fed BD + Cal had 8%–24% greater (*P* < 0.05) breaking strength than that of birds fed the other diets at week 6 (Table 5). However, there was no difference in other measured variables among the 4 treatment diets. In Exp. 2, birds fed BD + DHA + Cal had 19% higher (*P* < 0.05) BMD than birds fed BD + DHA diet and 13% higher (*P* < 0.05) total bone volume compared with birds fed BD and BD + DHA diets at week 6 (Table 6). Diets produced no significant effects on other measured variables including cortical BMD, cortical percentage bone volume, trabecular BMD, trabecular percentage bone volume, trabecular thickness, trabecular separation, or degree of anisotropy.

**Table 5:** Effects of supplementation of calcidiol and DHA-rich microalgal biomass on tibia bone properties of broiler chickens at week 6 in Exp. 1

Item	**BD**	**BD + Cal**	**BD + DHA**	**BD + Cal + DHA**	**SEM**	***P*-value**
Extension at maximum load, mm	2.13	2.12	2.06	2.21	0.10	0.79
Energy at maximum load, J	0.47^a^	0.56^b^	0.44^a^	0.49^ab^	0.04	0.02
Maximum slope, mm/N	0.09	0.05	0.05	0.04	0.01	0.06
Maximum load, N	500	515	514	520	31.5	0.97
Maximum extension, mm	2.37	2.49	2.44	2.30	0.17	0.14

BD = Corn-soybean basal diet, BD + Cal = BD + 10,000 IU calcidiol/kg of diet, BD + DHA = BD + 1% DHA-rich microalgal biomass, BD + Cal + DHA = BD + Cal + 1% DHA-rich microalgal biomass

^a,b^Means bearing the different superscripts in a row differ (*P* < 0.05)

Values are expressed as means of 6 birds/treatment and data were analyzed using two-way ANOVA

**Table 6:** Effects of supplementation of DHA-rich microalgal oil, EPA-rich microalgal biomass, and calcidiol on tibia bone properties of broiler chickens at week 6 in Exp. 2

Item	**BD**	**BD + DHA**	**BD + DHA+ EPA**	**BD + DHA+ Cal**	**BD + DHA+ EPA + Cal**	**SEM**	***P*-value**
Total BMD, g/cm^3^	0.27^ab^	0.26^a^	0.29^ab^	0.31^b^	0.30^ab^	0.01	0.04
Total bone volume, %	34^a^	34^a^	37^ab^	38^b^	36^ab^	1.5	0.04
Cortical BMD, g/cm^3^	0.60	0.58	0.61	0.66	0.65	0.03	0.38
Cortical bone Vvolume, %	100	100	100	100	100	0.001	0.20
Trabecular BMD, g/cm^3^	0.10	0.10	0.12	0.10	0.10	0.02	0.94
Trabecular bone volume, %	9.97	10.1	11.4	10.4	9.99	1.44	0.97
Trabecular thickness, mm	0.14	0.14	0.15	0.16	0.15	0.01	0.19
Trabecular separation, mm	1.20	1.31	1.22	1.24	1.36	0.11	0.52
Degree of anisotropy	1.70	1.72	1.65	1.66	1.63	0.05	0.72

*BMD* Bone mineral density

0–3 weeks: BD = Corn-soybean basal diet; BD + DHA = BD + 1.5 g of DHA oil/kg; BD + DHA + EPA = BD + DHA + 0.3 g/kg *Nannochloropsis* sp. CO18; BD + DHA + Cal = BD + DHA + 6000 IU calcidiol/kg; BD + DHA + EPA + Cal = BD + DHA + EPA (0.3 g/kg *Nannochloropsis* sp. CO18) + 6000 IU calcidiol/kg

4–6 Weeks: BD = Corn-soybean basal diet; BD + DHA = BD + 3.0 g DHA oil/kg; BD + DHA + EPA = BD + DHA + 0.6 g/kg *Nannochloropsis* sp. CO18; BD + DHA + Cal = BD + DHA + 12,000 IU calcidiol/kg; BD + DHA + EPA + Cal = BD + DHA + EPA (0.6 g/kg *Nannochloropsis* sp. CO18) + 12,000 IU calcidiol/kg

^a,b^Means bearing the different superscripts in a row differ (*P* < 0.05)

Values are expressed as means of 6 birds/treatment and data were analyzed using one-way ANOVA

## Discussion

Two consecutive experiments were conducted in the present study to determine responses of growth performance, tissue lipid profiles, and bone characteristics of broiler chickens to supranutrition of DHA and calcidiol during the full starter and grower periods. The sources and concentrations of these two nutrients were chosen based on our previous findings [9, 11] and literature [12, 13] to enrich them in the chicken tissues singularly or together. Indeed, supplementing these two nutrients into diets at the doses used in the present study led to 4–19-fold increases in DHA and 44%–52% increase in calcidiol contents of the liver, breast, and thigh (Kalia et al., manuscript in preparation). The most relevant finding for the broiler industry is that the high dietary supplemental levels of DHA and calcidiol exerted no negative effects on growth performance [8, 21], tissue lipid profiles, or bone characteristics. In contrast, those high supplementations actually exhibited moderate benefits to several measures. To our best knowledge, this was the first attempt to evaluate such effects of the two bioactive nutrients supplemented simultaneously at higher doses, although prior trials determined the effects of calcidiol [3, 4] and DHA-rich microalgal biomass or product [6, 22] singularly.

The enhanced BW, BWG, and gain:feed ratio by feeding birds with BD + Cal over BD in Exp. 1 were consistent with the reported improvements in body weight and feed efficiency resulted from feeding high doses of calcidiol to broiler chickens [13, 23] and breeders [24]. Interesting, birds fed BD + DHA + Cal had an increased feed intake and numerical improvements in BW and BWG. In Exp. 2, supplementing EPA into the BD + DHA diet further improved BW, BWG, and FI of broilers to be significantly higher than those of birds fed BD. In contrast, supplementing EPA into the BD + DHA + Cal diet improved only FI. This implies a potential benefit of supplemental EPA and a unique interaction between EPA and DHA and calcidiol on growth performance of chickens [25–27]. Long et al. [5] and Ribeiro et al. [28] reported that high doses of n-3 fatty acids in broiler diets improved BWG and feed conversion ratio. However, supplementing calcidiol alone decreased feed intake of chickens in two studies [13, 29]. Meanwhile, a number of studies indicated that broiler chickens or breeders responded well to dietary supplementations of 1400 to 2800 IU of cholecalciferol/kg diet [3, 4, 30] and 0.55 to 2.55 g DHA/kg diet [5–7]. Notably, Yarger et al. [8] found no evidence of renal calcification caused by supplemental 27,600 IU of calcidiol/kg, suggesting that our supplementation doses of calcidiol (6000 to 12,000 IU/kg) were within the safe range. Intriguingly, feeding broilers with 5520 IU of calcidiol/kg increased breast meat yield [31].

Because DHA and calcidiol are fat-soluble, bioactive nutrients that play major roles in regulating lipid metabolism [32, 33], we determined how the supranutrition of them intended for their enrichments in the chicken affected lipid profiles of plasma and tissues of chickens. Such effects will have both animal and human health implications. Whereas substantial changes in the plasma and tissue lipid profiles may reflect a metabolic shift to impair overall health status of broilers, the decreases in lipid contents of the tissues (muscles) may render chicken a more desired animal-sourced protein for human nutrition to reduce risks of chronic diseases. In Exp. 1, birds fed BD + Cal had lower plasma TC concentrations than those fed BD at week 6. This decrease agreed with the reported reduced plasma cholesterol levels in rodents fed high doses of calcidiol [34, 35]. Because 3-hydroxy-3-methylglutaryl coenzyme A (HMG-CoA) reductase catalyzes the major rate-limiting step in cholesterol biosynthesis [34], these supplemental calcidiol-mediated cholesterol decreases might be due to an inhibition of the enzyme by calcidiol [34, 35]. In Exp. 2, supplementing EPA into the BD + DHA diet, compared with BD, caused consistent decreases of concentrations of TGs in the plasma, liver, and breast and thigh muscles, concentrations of TC in the plasma, and concentrations of NEFAs in the breast muscle at week 6. At the same time, supplementing DHA into BD decreased plasma concentrations of NEFAs and the combined supplementations of DHA, EPA, and calcidiol decreased plasma concentrations of TC compared with BD. Previous studies [5, 36] showed hypocholesterolemic and hypotriacylglycerolemic effects of EPA and DHA via inhibiting squalene epoxidase enzyme [36, 37] and reducing very low-density lipoprotein (VLDL) synthesis and secretion [5, 38, 39], respectively. These regulations may be used to explain the decreases of TGs and TC by supplemental DHA and EPA in our study. The lack of any treatment effects on plasma and tissue lipid profiles of chickens at week 3 suggests that a longer time than 3 weeks was required for the dietary supplementations of DHA, EPA, and calcidiol to alter lipid metabolism in the broilers.

It is practically relevant to show an elevated energy at maximum load of the tibia bone from chickens fed BD + Cal compared with those fed BD in Exp. 1. This elevation implies that adding extra 10,000 IU of calcidiol/kg into the BD contained 300 IU of vitamin D_3_ improved tibia bone strength. Improvements in tibia bone strength and bone volume were produced by supplementing calcidiol at 2760 IU/kg [40] and 12,000 IU/kg [13], respectively, in the poultry diet. It will be interesting to find out if these improvements are still detectable when 10,000 to 12,000 IU calcidiol/kg is added to commercial diets supplemented with > 2000 IU of vitamin D_3_/kg. The lack of supplemental DHA alone or with calcidiol effect on tibia bone characteristics in Exp. 1 are consistent with outcomes of DHA supplementation on bone structural integrity and strength in several earlier studies [41–43]. In Exp.eriment 2, supplementing high levels of calcidiol into the BD + DHA diets appeared to improve total bone volume and total BMD, implying synergistic potential of DHA and calcidiol in improving bone mineral metabolism [44, 45] and reducing risk of bone fracture [46]. Because broiler chickens are among the most fast-growing animals and are susceptible to tibial dyschondroplasia (TD) that reduces the stability of leg bones and deteriorates the quality of meat from the legs [3], superanutrition of DHA and calcidiol may help not only enrichments of chicken with those nutrients but also prevention of TD and associated losses.

## Conclusions

Feeding broiler chickens with supranutritional levels of DHA, either from microalgal biomass or oil, and synthetic calcidiol from day 1 to day 42 of age produced no adverse effects on growth performance, plasma and tissue lipid profiles, or tibia characteristics. Instead, some of these singular or combined supplementations led to moderate beneficial responses of the three types of measures. Supplementing low levels of EPA into the BD + DHA diet or the BD + DHA + Cal diet resulted in rather consistent improvements in a number of those measures. There was a synergistic potential between supranutritions of DHA and calcidiol in improving tibial traits. Overall, it seems to be not only safe but also metabolically beneficial to supplement high levels of dietary DHA and calcidiol, much higher than the nutrient requirements, for biofortifying chicken with these bioactive nutrients.

## Supplementary Information

**Additional file 1:** **Table S1.** Composition of experimental diets used in starter period (Exp. 1). **Table S2.** Composition of experimental diets used in finisher period (Exp. 1). **Table S3.** Composition of experimental diets used in starter period (Exp. 2). **Table S4.** Composition of experimental diets used in finisher period (Exp. 2). **Table S5.** Effects of supplementation of calcidiol, DHA-rich microalgal biomass or oil, and EPA-rich microalgal biomass on body weight, feed intake, and gain: feed ratio in broiler chickens in Exp. 1 and 2 (0–3 weeks).

## Data Availability

All data generated or analyzed during this study are available from the corresponding author upon reasonablerequest.

## Abbreviations

BD: Basal diet
BMD: Bone mineral density
BWG: Body weight gain
Cal: Calcidiol (25(OH)D_3_)
DHA: Docosahexaenoic acid
EPA: Eicosapentaenoic acid
FI: Feed intake
NEFA: Non-esterified fatty acid
PL: Phospholipids
TC: Total cholesterol
TD: Tibial dyschondroplasia
TGs: Triglycerides
