# Supplementary material for: Supranutrition of microalgal docosahexaenoic acid and calcidiol improved growth performance, tissue lipid profiles, and tibia characteristics of broiler chickens
Source: J Anim Sci Biotechnol. 2023 Mar 16;14:27. doi: 10.1186/s40104-023-00842-3 (PMC10018906; doi:10.1186/s40104-023-00842-3)
Supplement: Supplementary file 1 — Additional file 1: Table S1. Composition of experimental diets used in starter period (Exp. 1). Table S2. Composition of experimental diets used in finisher period (Exp. 1). Table S3. Composition of experimental diets used in starter period (Exp. 2). Table S4. Composition of experimental diets used in finisher period (Exp. 2). Table S5. Effects of supplementation of calcidiol, DHA-rich microalgal biomass or oil, and EPA-rich microalgal biomass on body weight, feed intake, and gain: feed ratio in broiler chickens in Exp. 1 and 2 (0–3 weeks). [file 40104_2023_842_MOESM1_ESM.docx]

**Table S1** Composition of experimental diets used in starter period (Exp. 1)

| **Ingredient, g/kg** | **BD** | **BD+Cal** | **BD+DHA** | **BD+Cal+DHA** |
| --- | --- | --- | --- | --- |
| Corn | 579 | 579 | 579 | 579 |
| Soybean Meal | 342 | 342 | 342 | 342 |
| DHA Algae^1^ | 0.00 | 0.00 | 10.0 | 10.0 |
| Calcidiol, 62.5 Rovimix^2^ | 0.00 | 0.91 | 0.00 | 0.91 |
| Corn Oil | 34.0 | 34.0 | 27.5 | 27.5 |
| Dicalcium Phosphate | 19.6 | 19.6 | 19.6 | 19.6 |
| Limestone | 6.30 | 6.30 | 6.30 | 6.30 |
| NaCl | 4.70 | 4.70 | 4.70 | 4.70 |
| Methionine | 2.10 | 2.10 | 2.10 | 2.10 |
| Lysine | 0.50 | 0.50 | 0.50 | 0.50 |
| Celite | 7.30 | 6.40 | 3.80 | 2.89 |
| Vitamin/Mineral Mix^3^ | 4.50 | 4.50 | 4.50 | 4.50 |
| Nutritive Value |  |  |  |  |
| ME, Kcal/kg | 3092 | 3077 | 3077 | 3077 |
| Crude Protein, % | 20.7 | 20.5 | 20.6 | 20.6 |
| Crude Fat, % | 5.76 | 5.75 | 5.60 | 5.60 |
| Ca, % | 1.00 | 0.98 | 0.98 | 0.98 |
| P, % | 0.76 | 0.71 | 0.71 | 0.71 |
| Na, % | 0.20 | 0.20 | 0.20 | 0.20 |

BD = Corn-soybean basal diet; BD+Cal = BD + 10000 IU calcidiol/kg of diet; BD+DHA = BD + 1% DHA-rich microalgal biomass; BD+Cal+DHA = BD+Cal + 1% DHA-rich microalgal biomass

^1^DHA Algae = *Aurantiochytrium* biomass

^2^Rovimix HY-D Premix 62.5

^3^The vitamin/mineral mix contained vitamin A 2250 IU/kg, vitamin D 300 IU/kg, vitamin E 20 IU/kg, vitamin K 0.752 mg/kg, biotin 0.225 mg/kg, choline 1950 mg/kg, folacin 0.825, niacin 52.5 mg/kg, pantothenic acid 15 mg/kg, riboflavin 5.40 mg/kg, thiamine 2.70 mg/kg, vitamin B_6_ 5.25 mg/kg, vitamin B_12_ 0.015 mg/kg, copper 12 mg/kg, iodine 0.53 mg/kg, manganese 91.08 mg/kg, zinc 60.24 mg/kg, selenium 0.20 mg/kg, and iron 80 mg/kg

**Table S2** Composition of experimental diets used in finisher period (Exp. 1)

| **Ingredient, g/kg** | **BD** | **BD+Cal** | **BD+DHA** | **BD+Cal+DHA** |
| --- | --- | --- | --- | --- |
| Corn | 590 | 590 | 589 | 588 |
| Soybean Meal | 330 | 330 | 328 | 328. |
| DHA Algae^1^ | 0.00 | 0.00 | 10.0 | 10.0 |
| Calcidiol, 62.5 Rovimix^2^ | 0.00 | 0.91 | 0.00 | 0.91 |
| Corn Oil | 44.0 | 44.0 | 38.0 | 38.0 |
| Dicalcium Phosphate | 15.3 | 15.30 | 15.3 | 15.3 |
| Limestone | 8.40 | 8.40 | 8.40 | 8.40 |
| NaCl | 4.70 | 4.70 | 4.70 | 4.70 |
| Methionine | 1.10 | 1.10 | 1.10 | 1.10 |
| Threonine | 0.70 | 0.70 | 0.70 | 0.70 |
| Celite | 1.30 | 0.39 | 0.30 | 0.00 |
| Vitamin/Mineral Mix^3^ | 4.50 | 4.50 | 4.50 | 4.50 |
| Nutritive Value |  |  |  |  |
| ME, Kcal/kg | 3170 | 3170 | 3167 | 3167 |
| Crude Protein, % | 20.0 | 20.0 | 20.0 | 20.0 |
| Crude Fat, % | 6.77 | 6.77 | 6.66 | 6.66 |
| Ca, % | 0.90 | 0.90 | 0.90 | 0.90 |
| P, % | 0.69 | 0.67 | 0.67 | 0.67 |
| Na, % | 0.20 | 0.20 | 0.20 | 0.20 |

BD = Corn-soybean basal diet; BD+Cal = BD + 10000 IU calcidiol/kg of diet; BD+DHA = BD + 1% DHA-rich microalgal biomass; BD+Cal+DHA = BD+Cal + 1% DHA-rich microalgal biomass

^1^DHA Algae = *Aurantiochytrium* biomass

^2^Rovimix HY-D Premix 62.5

^3^The vitamin/mineral mix contained vitamin A 2250 IU/kg, vitamin D 300 IU/kg, vitamin E 20 IU/kg, vitamin K 0.752 mg/kg, biotin 0.225 mg/kg, choline 1950 mg/kg, folacin 0.825, niacin 52.5 mg/kg, pantothenic acid 15 mg/kg, riboflavin 5.40 mg/kg, thiamine 2.70 mg/kg, vitamin B_6_ 5.25 mg/kg, vitamin B_12_ 0.015 mg/kg, copper 12 mg/kg, iodine 0.53 mg/kg, manganese 91.08 mg/kg, zinc 60.24 mg/kg, selenium 0.20 mg/kg, and iron 80 mg/kg

#### **Table S3** Composition of experimental diets used in starter period (Exp. 2)

| **Ingredients, g/kg** | **BD** | **BD+DHA** | **BD+DHA**  **+EPA** | **BD+DHA**  **+Cal** | **BD+DHA**  **+EPA+Cal** |
| --- | --- | --- | --- | --- | --- |
| Corn | 501 | 501 | 505 | 501 | 505 |
| Soybean meal | 405 | 405 | 390 | 405 | 390 |
| DHA Oil | 0.00 | 3.30 | 3.30 | 3.30 | 3.30 |
| Corn Oil | 34.00 | 30.7 | 30.7 | 30.7 | 30.7 |
| *N. oceanica* | 0.00 | 0.00 | 19.0 | 0.00 | 19.0 |
| Calcidiol, 62.5 Rovimix^1^ | 0.00 | 0.00 | 0.00 | 1.09 | 1.09 |
| Dicalcium Phosphate | 18.1 | 18.1 | 18.0 | 18.1 | 18.0 |
| Limestone | 14.00 | 14.0 | 14.5 | 14.0 | 14.5 |
| NaCl | 5.00 | 5.00 | 4.20 | 5.00 | 4.20 |
| Met | 3.00 | 3.00 | 2.90 | 3.00 | 2.90 |
| *L*-Lysine-HCl | 0.00 | 0.00 | 0.00 | 0.00 | 0.00 |
| *L*-Threonine | 0.80 | 0.80 | 0.79 | 0.80 | 0.79 |
| Cellite | 12.7 | 12.7 | 5.73 | 11.61 | 4.64 |
| Vit/min mix^2^ | 5.88 | 5.88 | 5.88 | 5.88 | 5.88 |
| **Nutritive value** |  |  |  |  |  |
| ME, Kcal/kg | 2968 | 2968 | 2982 | 2968 | 2982 |
| Crude Protein, % | 23.0 | 23.0 | 23. | 23.0 | 23.1 |
| Crude Fat, % | 5.58 | 5.25 | 5.39 | 5.25 | 5.39 |
| Ca, % | 1.00 | 1.00 | 1.02 | 1.00 | 1.02 |
| P, % | 0.78 | 0.78 | 0.79 | 0.78 | 0.79 |
| Na, % | 0.20 | 0.20 | 0.22 | 0.20 | 0.22 |

0–3 Weeks: BD = Corn-soybean basal diet; BD+DHA= BD + 1.5 g of DHA oil/kg; BD+DHA+EPA = BD+DHA + 0.3 g/kg *Nannochloropsis* sp. CO18; BD+DHA+Cal = BD+DHA + 6000 IU calcidiol/kg; BD+DHA+EPA+Cal = BD+DHA+EPA (0.3 g/kg *Nannochloropsis* sp. CO18) + 6000 IU calcidiol/kg

^1^Rovimix HY-D Premix 62.5

^2^The vitamin/mineral mix contained vitamin A 2250 IU/kg, vitamin D 300 IU/kg, vitamin E 20 IU/kg, vitamin K 0.75 mg/kg, biotin 0.225 mg/kg, choline 2600 mg/kg, folacin 0.825 mg/kg, niacin 52.50 mg/kg, pantothenic acid 15 mg/kg, riboflavin 5.4 mg/kg, thiamine 2.07 mg/kg, vitamin B6 5.25 mg/kg, vitamin B12 0.015 mg/kg, copper 12 mg/kg, iodine 0.53 mg/kg, manganese 91.08 mg/kg, zinc 60.24 mg/kg, selenium 0.20 mg/kg, and iron 80 mg/kg

**Table S4** Composition of experimental diets used in finisher period (Exp. 2)

| **Ingredients, g/kg** | **BD** | **BD+DHA** | **BD+DHA**  **+EPA** | **BD+DHA**  **+Cal** | **BD+DHA**  **+EPA+Cal** |
| --- | --- | --- | --- | --- | --- |
| Corn | 581 | 581 | 580 | 581 | 580 |
| Soybean meal | 330 | 330 | 304 | 330 | 304 |
| DHA Oil | 0.00 | 6.60 | 6.60 | 6.60 | 6.60 |
| Corn Oil | 30.0 | 23.4 | 23.4 | 23.4 | 23.4 |
| *N. oceanica* | 0.00 | 0.00 | 38.0 | 0.00 | 38.0 |
| Calcidiol, 62.5 Rovimix^1^ | 0.00 | 0.00 | 0.00 | 2.18 | 2.18 |
| Dicalcium Phosphate | 13.6 | 13.6 | 13.6 | 13.6 | 13.6 |
| Limestone | 14.0 | 14.0 | 14.0 | 14.0 | 14.0 |
| NaCl | 5.00 | 5.00 | 5.00 | 5.00 | 5.00 |
| Met | 1.80 | 1.80 | 1.63 | 1.80 | 1.63 |
| *L*-Lysine-HCl | 0.60 | 0.60 | 1.00 | 0.60 | 1.00 |
| *L*-Threonine | 1.00 | 1.00 | 1.00 | 1.00 | 1.00 |
| Cellite | 15.9 | 15.9 | 5.79 | 13.7 | 3.61 |
| Vit/min mix^2^ | 5.88 | 5.88 | 5.88 | 5.88 | 5.88 |
| **Nutritive value** |  |  |  |  |  |
| ME, Kcal/kg | 3025 | 3025 | 3030 | 3025 | 3030 |
| Crude Protein, % | 20.0 | 20.0 | 20.5 | 20.0 | 20.5 |
| Crude Fat, % | 5.35 | 4.69 | 4.94 | 4.69 | 4.94 |
| Ca, % | 0.90 | 0.90 | 0.90 | 0.90 | 0.90 |
| P, % | 0.66 | 0.66 | 0.68 | 0.66 | 0.68 |
| Na, % | 0.20 | 0.20 | 0.30 | 0.20 | 0.30 |

4–6 Weeks: BD = Corn-soybean basal diet; BD+DHA= BD + 3.0 g DHA oil/kg; BD+DHA+EPA = BD+DHA + 0.6 g/kg *Nannochloropsis* sp. CO18; BD+DHA+Cal = BD+DHA + 12000 IU calcidiol/kg; BD+DHA+EPA+Cal = BD+DHA+EPA (0.6 g/kg *Nannochloropsis* sp. CO18) + 12000 IU calcidiol/kg

^1^Rovimix HY-D Premix 62.5

^2^The vitamin/mineral mix contained vitamin A 2250 IU/kg, vitamin D 300 IU/kg, vitamin E 20 IU/kg, vitamin K 0.75 mg/kg, biotin 0.225 mg/kg, choline 2600 mg/kg, folacin 0.825 mg/kg, niacin 52.50 mg/kg, pantothenic acid 15 mg/kg, riboflavin 5.4 mg/kg, thiamine 2.07 mg/kg, vitamin B_6_ 5.25 mg/kg, vitamin B_12_ 0.015 mg/kg, copper 12 mg/kg, iodine 0.53 mg/kg, manganese 91.08 mg/kg, zinc 60.24 mg/kg, selenium 0.20 mg/kg, and iron 80 mg/kg

**Table S5** Effects of supplementation of calcidiol, DHA-rich microalgal biomass or oil, and EPA-rich microalgal biomass on body weight, feed intake, and gain: feed ratio in broiler chickens in Experiments 1 and 2 (0–3 weeks)

| **Exp. 1** | | | | | | | |
| --- | --- | --- | --- | --- | --- | --- | --- |
| **Parameters** | **BD** | **BD+Cal** | **BD+DHA** | **BD+Cal+DHA** | **SEM** | ***P*-value** |  |
| BW 0 d, g/chick | 41.9 | 41.8 | 41.8 | 41.7 | 0.170 | 0.85 |  |
| BW 0-3 weeks, g/chick | 865 | 880 | 842 | 867 | 15.15 | 0.39 |  |
| BWG 0-3 weeks, g/chick | 823 | 838 | 800 | 825 | 17.2 | 0.51 |  |
| FI 0-3 weeks, g/chick | 1160 | 1140 | 1100 | 1150 | 34.0 | 0.18 |  |
| Gain: Feed 0-3 weeks | 0.710 | 0.730 | 0.730 | 0.720 | 0.016 | 0.34 |  |
| **Exp. 2** | | | | | | | |
|  | **BD** | **BD+DHA** | **BD+DHA**  **+EPA** | **BD+DHA**  **+Cal** | **BD+DHA**  **+EPA+Cal** | **SEM** | ***P*-value** |
| BW 0 d, g/chick | 42.3 | 42.3 | 42.4 | 42.4 | 42.4 | 0.07 | 0.64 |
| BW 0-3 weeks, g/chick | 566 | 540 | 662 | 522 | 555 | 30.9 | 0.08 |
| BWG 0-3 weeks, g/chick | 523 | 497 | 620 | 479 | 512 | 27.2 | 0.07 |
| FI 0-3 weeks, g/chick | 819 | 777 | 911 | 839 | 923 | 30.4 | 0.10 |
| Gain: Feed 0-3 weeks | 0.640 | 0.640 | 0.680 | 0.570 | 0.560 | 0.03 | 0.08 |

BW: Body weight; BWG: Body weight gain; FI: Feed intake

Experiment 1; BD = Corn-soybean basal diet; BD+Cal = BD + 10000 IU calcidiol/kg of diet; BD+DHA = BD + 1% DHA-rich microalgal biomass; BD+Cal+DHA = BD+Cal + 1% DHA-rich microalgal biomass.

Experiment 2; 0-3 Weeks: BD = Corn-soybean basal diet; BD+DHA= BD + 1.5 g of DHA oil/kg; BD+DHA+EPA = BD+DHA + 0.3 g/kg *Nannochloropsis* sp. CO18; BD+DHA+Cal = BD+DHA + 6000 IU calcidiol/kg; BD+DHA+EPA+Cal = BD+DHA+EPA (0.3 g/kg *Nannochloropsis* sp. CO18) + 6000 IU calcidiol/kg

Means bearing the different superscripts (a, b) in a row differ (*P* < 0.05)

Data are expressed as means (*n* = 6 cages and 6 birds/cage) and were analyzed by two-way and one-way ANOVA in Experiments 1 and 2, respectively
